# Supplementary material for: Double Mutations in Succinate Dehydrogenase Are Involved in SDHI Resistance in Corynespora cassiicola
Source: Microorganisms. 2022 Jan 9;10(1):132. doi: 10.3390/microorganisms10010132 (PMC8779870; doi:10.3390/microorganisms10010132)
Supplement: Supplementary file 1 [file microorganisms-10-00132-s001.zip › microorganisms-1536717-supplementary.pdf]

Supplementary Materials:

Table S1. List of cases of SDHI-resistant plant fungal pathogens, their origins, and the double mutations found to be associated with SDHI resistance.

Species name	Reported host	Origin	Resistance mechanism (Subunit-mutation)	Refer-ence
Mycosphaerella gramini-cola	Wheat	Field	C-N86S+B-N225T, C-N86S+ C-T79N and C-N86S+C-L85P	1
Mycosphaerella gramini-cola	Wheat	Lab	B-S221P+C-R54G, B-H267Y+C-N86S, B-D166G+D-D129G, B-P155L+B-H267Y, C-L85P+D-V96A	3
Alternaria solani	Potato	Field	B-H278Y+C-H134R, B-H278R+C-H134R, D-T28A+D-A47T, B-277R+D-133R	2, 4
Alternaria alternata	Potato	Field	B-H277Y+C-H134R and B-H277R+C-H134R	2, 5
Alternaria alternata	Murcott tangor	Field	C-G84S+C-P130A	6

Reference

1 Minutes and recommendations of the SDHI Working Group from 21st of January 2021 are now available. FRAC

2 Landschoot, S.; Carrette, J.; Vandecasteele M. Boscalid-resistance in Alternaria alternata and Alternaria solani populations: An emerging problem in Europe. *Crop Protection* **2017**, 92, 49-59.

3 Fraaije, B.A.; Bayon, C.; Atkins, S.; Cools, H.J.; Lucas, J.A.; Fraaije, M.W. Risk assessment studies on succinate dehydrogenase inhibitors, the new weapons in the battle to control Septoria leaf blotch in wheat. *Mol. Plant Pathol.* **2012**, 13, 263-75.

4 Miles, T.D.; Miles, L.A.; Fairchild, K.L.; Wharton, P.S. Screening and characterization of resistance to succinate dehydrogenase inhibitors in Alternaria solani. *Plant Pathology* **2014**, 63, 155-64.

5 Avenot, H.F.; Van Den Biggelaar, H.; Morgan, D.P.; Moral, J.; Joosten, M.; Michailides, T.J. Sensitivities of Baseline Isolates and Boscalid-Resistant Mutants of Alternaria alternata from Pistachio to Fluopyram, Penthiopyrad, and Fluxapyroxad. *Plant Dis.* **2014**, 98, 197-205.

6 Vega, B.; Dewdney, M.M. Sensitivity of Alternaria alternata from Citrus to Boscalid and Polymorphism in Iron-Sulfur and in Anchored Membrane Subunits of Succinate Dehydrogenase. *Plant Dis.* **2015**, 99, 23

Table S2. Isolates used in this study.

Isolate	Genotype	Mutation		Origin	Location
		sdhB	*sdhD*		
SD1	WT	WT	WT	Field	Shandong
R25	B-H278R	H278R	WT	SD1	-
R34	B-H278R	H278R	WT	SD1	-
Y25	B-H278Y	H278Y	WT	SD1	-
V43	B-I280V	I280V	WT	SD1	-
V44	B-I280V	I280V	WT	SD1	-
E14	D-D95E	WT	D95E	SD1	-
H30	D-H105R	WT	H105R	SD1	-
V39	D-G109V	WT	G09V	SD1	-
ER149	B-H278R+D-D95E	H278R	D95E	E14	-
EV31	B-I280V+D-D95E	I280V	D95E	E14	-
EY54	B-H278Y+D-D95E	H278Y	D95E	E14	-
VR93	B-H278R+D-G109V	H278R	G109V	V39	-
VV3	B-I280V+D-G109V	I280V	G109V	V39	-
VY3	B-H278Y+D-G109V	H278Y	G109V	V39	-
HV115	B-I280V+D-H105R	I280V	H105R	H30	-

Table S3. Primers and probes used in this study

Primer	Sequence (5′-3')	Use
P1	CACTCTTCTTCGCCATCC	Amplify the *sdhB* gene of *C. cassiicola* (1422 bp)
P2	CATCACACTCACGGTCAC	
P3	CTGCGATTGGGCTTTCTAC	Identification of the *sdhB* +*Trpc*+*neo* cassette integrated at the left junction (2575 bp)
P4	TGTCCTCGTTCCTGTCTGC	
P5	GGGACTGGCTGCTATTGG	Identification of the *sdhB* +*Trpc*+*neo* cassette integrated at the right junction (2791 bp)
P6	CCTCCGAGGTCGAGGATTT	
P7	CGAGAGATGAAGAATCGGTA	Confirm the homozygosity of the *sdhB* +*Trpc*+*neo* cassette inte-grated at the left junction (3256/2091 bp)
P8	GACCAATCCACTCCGTTA	
P9	AAGACCTTCCACATCTACC	Confirm the homozygosity of the *sdhB* +*Trpc*+*neo* cassette inte-grated at the right junction (2192/1027 bp)
P10	GGGTGACCAGAACAGTAT	
P11	CTGCGATTGGGCTTTCTA	Amplification of the upstream region and *sdhB* gene fragment (2352 bp)
P12	CAATATCATCTTCTGTCGACCTACGTGAAAGCCATGCTC	
P13	CGCCTTCTTGACGAGTTCTTCTGAAGTCGTTGCTGAATGGGT	Amplification of the region downstream of the *sdhB* gene (1462 bp)
P14	GGACTCGCACTTCCTCAA	
P15	GTCGACAGAAGATGATATTG	Amplification of the *Trpc*+*neo* gene (1165 bp)
P16	TCAGAAGAACTCGTCAAGAAGGCG	
P17	CGGACATGTCTAGCAAAGTC	Amplification of the *sdhB* +*Trpc*+*neo* gene replacement cassette (4484 bp)
P18	GCTTCTAACAAGTCCCGT	
P19	TTGAGATGGCTGTGGATATG	Amplification of the probe for *sdhB* (858 bp)

P20	AGATGTGGAAGGTCTTGGT	
P21	TCACCGTCATTGACGCCC	Amplification of the *EF1-α* gene for quantitative real-time poly-
P22	CGGCAGCGATAATGAGGATAG	merase chain reaction (96 bp)
P23	GCTGGACCTGAACAAGACCG	Amplification of the *sdhB* gene for quantitative real-time poly-
P24	GATGCGGCAAAGACAGG	merase chain reaction (166 bp)
P25	CTACCACTGGAGCTTCGAGAGGGC	Amplification of the *sdhD* gene for quantitative real-time poly-
P26	CGCTTGGCAGGGAAGTAGTCA	merase chain reaction (174 bp)
P27	GAAACATGCACGAGGACG	Amplification of the *sdhA* gene for quantitative real-time poly-
P28	TCGGTACGTGAGAATGGG	merase chain reaction (157 bp)
P29	TGGTCCAGCGGAGAGCGG	Amplification of the *sdhC* gene for quantitative real-time poly-
P30	TGGGGGCAGCCAGGTAGG	merase chain reaction (213 bp)

Table S4. Fitness of each mutant with respect to WT, for each fitness component test.

SdhB/D substitution		B-H278R			B-H278Y			B-I280V			D-D95E			D-H105R		
Fitness component		+	=	-	+	=	-	+	=	-	+	=	-	+	=	-
SDH activity		0	0	1	0	0	1	0	1	0	0	1	0	0	0	1
Conidia	production	0	0	1	0	0	1	0	0	1	0	1	0	0	1	0
	germination	0	1	0	0	1	0	0	0	1	0	1	0	0	0	1
Mycelial growths		0	5	0	0	1	4	1	3	1	1	4	0	0	4	1
Pathogenicity		0	0	1	0	1	0	0	1	0	0	1	0	0	1	0
Environment stresses	osmotic stress	0	0	3	0	2	1	0	3	0	0	0	3	1	2	0
	oxidative stress	0	1	0	0	1	0	1	0	0	0	1	0	0	1	0
	cell wall damage	0	2	0	0	1	1	1	0	1	1	0	1	0	2	0
	salicylhydroxamic acid	0	1	0	0	1	0	0	1	0	0	1	0	0	1	0
Total [0,16]		0	10	6	0	8	8	3	9	4	2	10	4	1	12	3
%		0	63	38	0	50	50	19	56	25	13	63	25	6	75	19
Fitness score		25			0			68.75			62.5			68.75		

SdhB/D substitution		D-G109V			B-I280V+D-D95E			B-I280V+D-H105R			B-I280V+D-G109V		
Fitness component		+	=	-	+	=	-	+	=	-	+	=	-
SDH activity		0	0	1	0	1	0	0	0	1	0	0	1
Conidia	production	1	0	0	1	0	0	0	1	0	0	1	0
	germination	0	1	0	0	1	0	0	1	0	0	0	1
Mycelial growths		0	5	0	0	4	1	0	3	2	0	5	0
Pathogenicity		0	1	0	0	1	0	0	1	0	0	1	0
Environment stresses	osmotic stress	0	2	1	0	2	1	1	2	0	0	2	1
	oxidative stress	0	1	0	0	1	0	0	1	0	1	0	0
	cell wall damage	1	1	0	0	2	0	0	2	0	1	1	0
	salicylhydroxamic acid	0	1	0	0	1	0	0	1	0	0	1	0
Total [0,16]		2	12	2	1	13	2	1	12	3	2	11	3
%		13	75	13	6.25	81.25	12.5	6.25	75	18.75	12.5	68.8	18.75
Fitness score		87.5			81.25			68.75			75		

SdhB/D substitution		B-H278R+D-G109V			B-H278R+D-D95E			B-H278Y+D-G109V			B-H278Y+D-D95E		
Fitness component		+	=	-	+	=	-	+	=	-	+	=	-
SDH activity		0	0	1	0	1	0	0	0	1	0	0	1
Conidia	production	0	0	1	0	1	0	0	0	1	1	0	1
	germination	0	0	1	0	1	0	0	1	0	0	1	0
Mycelial growths		0	1	4	0	4	1	0	3	2	0	3	2
Pathogenicity		0	1	0	0	0	1	0	1	0	0	0	1
Environment stresses	osmotic stress	1	2	0	0	3	0	1	2	0	0	0	3
	oxidative stress	1	0	0	0	1	0	0	1	0	1	0	0
	cell wall damage	1	0	1	0	1	1	1	0	1	0	0	2
	salicylhydroxamic acid	0	0	1	0	1	0	0	1	0	0	0	1
Total [0,16]		3	4	9	0	13	3	2	9	5	2	4	11
%		18.8	25	56.25	0	81.25	18.75	12.5	56.25	31.25	12.5	25	68.75
Fitness score		6.25			62.5			50			-18.75		

For each fitness component test of the double mutations, a point is assigned in the +, = or - category, depending on the test result in comparison to the WT.' +' means that the fitness of the double mutations was significantly higher than WT. conversely, '-' means that more fitness costs were detected.' =' represents similar results were found compared to WT. The position of the numbers indicates the result of comparing the transformants to the WT.   The overall fitness score for each mutant was calculated as the percentage of scores in the = category, minus the percentage of scores in the - category, plus two times the percentage of scores in the + category (100% is the WT score). In other words, we chose a weight of -1 for the - category, 1 for the = category and 2 for the + category (Shi et al., 2021).

Table S5. Correlation analysis between SDH activity and expression of the *sdhA* gene

**Spearman's correlations**		***sdhA* gene expression**
SDH enzyme activity	Correlation coefficient (r)	-0.704
	Significance test (P)	0.007
	Number	14
